# Advantages of Metabolomics-Based Multivariate Machine Learning to Predict Disease Severity: Example of COVID

**DOI:** 10.3390/ijms252212199

**Published:** 2024-11-13

**Authors:** Maryne Lepoittevin, Quentin Blancart Remaury, Nicolas Lévêque, Arnaud W. Thille, Thomas Brunet, Karine Salaun, Mélanie Catroux, Luc Pellerin, Thierry Hauet, Raphael Thuillier

**Affiliations:** 1 Inserm Unit Ischémie Reperfusion, Métabolisme et Inflammation Stérile en Transplantation (IRMETIST), UMR U1313, F-86073 Poitiers, France; lepoittevin.maryne@gmail.com (M.L.); luc.pellerin@univ-poitiers.fr (L.P.); thierry.hauet@univ-poitiers.fr (T.H.); 2 Faculty of Medicine and Pharmacy, University of Poitiers, F-86073 Poitiers, France; 3 UMR CNRS 7285, Institut de Chimie des Milieux et Matériaux de Poitiers (IC2MP), University of Poitiers, 4 rue Michel-Brunet, TSA 51106, F-86073 Poitiers cedex 9, France; quentin.blancart.remaury@univ-poitiers.fr; 4 LITEC, CHU de Poitiers, Laboratoire de Virologie et Mycobactériologie, Université de Poitiers, 2 r Milétrie, F-86000 Poitiers, France; nicolas.leveque@chu-poitiers.fr; 5 Intensive Care Medicine Department, CHU Poitiers, F-86021 Poitiers, France; arnaud.thille@chu-poitiers.fr (A.W.T.); karine.salaun@chu-poitiers.fr (K.S.); 6 Geriatric Medicine Department, CHU Poitiers, F-86021 Poitiers, France; thomas.brunet@chu-poitiers.fr; 7 Internal Medicine and Infectious Disease Department, CHU Poitiers, F-86021 Poitiers, France; melanie.catroux@chu-poitiers.fr; 8 Biochemistry Department, CHU Poitiers, F-86021 Poitiers, France

**Keywords:** predictive algorithm, metabolomics, machine learning, COVID-19

## Abstract

The COVID-19 outbreak caused saturations of hospitals, highlighting the importance of early patient triage to optimize resource prioritization. Herein, our objective was to test if high definition metabolomics, combined with ML, can improve prognostication and triage performance over standard clinical parameters using COVID infection as an example. Using high resolution mass spectrometry, we obtained metabolomics profiles of patients and combined them with clinical parameters to design machine learning (ML) algorithms predicting severity (herein determined as the need for mechanical ventilation during patient care). A total of 64 PCR-positive COVID patients at the Poitiers CHU were recruited. Clinical and metabolomics investigations were conducted 8 days after the onset of symptoms. We show that standard clinical parameters could predict severity with good performance (AUC of the ROC curve: 0.85), using SpO2, first respiratory rate, Horowitz quotient and age as the most important variables. However, the performance of the prediction was substantially improved by the use of metabolomics (AUC = 0.92). Our small-scale study demonstrates that metabolomics can improve the performance of diagnosis and prognosis algorithms, and thus be a key player in the future discovery of new biological signals. This technique is easily deployable in the clinic, and combined with machine learning, it can help design the mathematical models needed to advance towards personalized medicine.

## 1. Introduction

Emergency services are often saturated, as was the case during the COVID-19 outbreak, with significant consequences for healthcare providers, from resource allocations to prioritization of patients [1]. A necessary step for such triage is correct patient outcome prediction. While recent studies have demonstrated the benefits of newer techniques such as Forced Oscillation Technique (FOT) [2], as well as novel biomarkers such as plasma KL-6 levels combined with chest radiographic severity grade (RSG) [3], the limited number of tools at the clinician’s disposal condemn the healthcare staff to react to the symptoms rather than anticipate them. It is therefore crucial to develop and validate new tools to help design the care plan for each patient [4].

One major hurdle in creating such a tool is the multifaceted nature of the encountered pathologies, the disparity of the symptoms, their apparent severity and the speed at which an apparently mild ailment can progress to severe outcomes.

To apprehend the multifactorial nature of such diseases, researchers have deployed machine learning-based strategies that can easily integrate a variety of clinical parameters. Indeed, such data mining technologies demonstrate an interesting ability of predicting the outcome; for instance, with COVID, measurable benefits for the patients and the healthcare system were shown [5], with several authors demonstrating the prognostic value of blood parameters when risk stratification is optimized by machine learning, combining co-morbidities with blood parameters [6], or only using blood gas parameters which can be obtained rapidly with delocalized biochemistry automata [7]. Usually, ML is capable of producing reliable risk scores for hospitalization or mortality based on typically measured blood parameters [8,9,10].

On the other hand, ML is sometimes not capable of producing reliable and reproducible scoring algorithms, as shown in a recent systematic review of more than 400 ML models using radiographs and scans in COVID-positive patients [11]. Hence, it is important not to limit the source of data to train the model. One important new source of data is high resolution mass spectrometry to perform metabolomics analysis. This permits the extraction of numerous biological signals from a small volume of sample and thus optimizes valorization of the patient’s fluids. Regarding COVID, these studies have identified a wide variety of biomarkers [12], with many studies identifying dysregulation of amino acids, and others focusing on patient lipidomes [13,14,15,16]. Indeed, authors have recently shown a good degree of performance from prediction algorithms using metabolomics data from saliva [17,18] or plasma [19,20].

A synthetic literature review is proposed in the Supplementary Materials.

The objective of our study was to test the hypothesis that metabolomics, combined with ML, can improve COVID severity prediction. To this end, we compared the performance of models issued from blood parameters highlighted in the literature with models issued from a dataset combining such parameters with results from a metabolomics screening.

## 2. Results

### 2.1. Patients Clinical and Bloodwork Parameters

A total of 69 patients were included in the study, of which 5 withdrew consent (Supplementary Figure S1). No patients were under artificial ventilation at the time of blood sample collection. The severity of COVID infection was determined by the necessity for mechanical ventilation during patient care. Such a parameter is indeed acknowledged in the Institute for Health and Care Excellence guidelines for the use of dexamethasone in COVID-positive patients, a recommendation also formulated by the World Health Organization [21]. The non-severe patients were slightly more numerous that the severe patients (35–29, respectively).

We explored blood parameters highlighted as pertinent for COVID severity prediction by the literature (detailed list and references of the 39 parameters are displayed in Supplementary Table S1). Of these, only 5 were statistically different between the populations, with severe patients surprisingly demonstrating a higher proportion of younger people, a higher respiratory rate and SpO2. Severe patients also showed significantly lower coagulation time as well as lower serum albumin.

### 2.2. Predicting COVID Severity with Clinical and Bloodwork Parameters and/or Metabolomics

**Figure 1:**
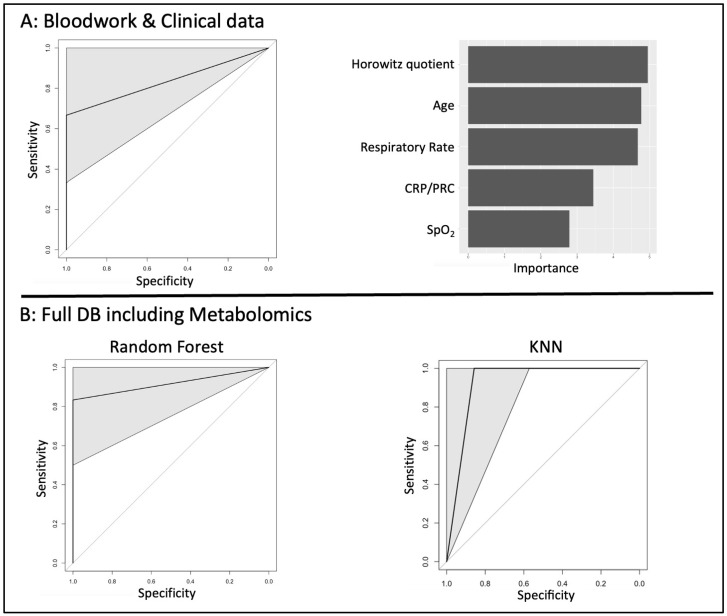
COVID-19 severity predictive algorithm performance. Several machine learning classifiers were used to attempt to build a predictive algorithm for COVID-19 severity 8 days after the onset of symptoms, using two different datasets. Representative ROC curves and main contributing variables are shown. (**A**) Performance and contributive variables of the Random Forest model built from the database containing bloodwork and clinical data only. (**B**) Performance and contributive variables of the Random Forest model built from the dataset containing bloodwork, clinical data and metabolomics screening. ROC curves including 95% confidence intervals were drawn using the pROC package and a bootstrapping strategy.

To test the predictive potential of the 39 clinical parameters, we conducted machine learning-based approaches to generate predictive algorithms. Of all the algorithms tested, Random Forest showed the most relevant performances across all indicators, however with a large confidence interval. SpO2, first respiratory rate, Horowitz quotient, age and CRP were the most important variables (Supplementary Table S2, Top, and Figure 1A).

Then, we explored the ability of metabolomics data alone to generate a predictive algorithm, (Table 1, Middle) showing that, on its own, it was unsuccessful in providing a performant discrimination between severe and non-severe patients.

However, combining both clinical and bloodwork parameters with results from the metabolomic study (Table 1, Bottom and Figure 1B), we showed that both Random Forest and KNN had significant performances, with higher AUC of the ROC curves compared to the clinical and bloodwork parameters model, with tighter confidence intervals. Extracting the parameters used for these algorithms revealed that the top ten included only metabolites (Supplementary Table S3).

Hence, metabolomics substantially improved the predictive performance of typical clinical parameters, showing a high potential of complementarity between the techniques.

## 3. Discussion

Herein, we conducted a small-scale clinical study to evaluate the potential of using LC-MS HRMS metabolomics to improve disease prediction. COVID-infection was used as an example of a pathology requiring such prognostication, using the need for mechanical ventilation as severity [21].

Our results show that, indeed, while parameters alone have good predictive performance, combination with metabolomics substantially improves prediction and precision of infection severity in COVID-positive patients.

Considering the later algorithm was based mainly on metabolites, such improvement in the performance in terms of patient discrimination demonstrates the potential of metabolomics regarding decision making at the bedside, confirming recent conclusions in the literature [22].

We tested several classifiers in our ML strategy and differing results demonstrated the wisdom of this approach, suggesting that any ML-based approach should propose several classifiers each representing a different way to analyze data. The robustness of Random Forest [23] in regard to its capability to extract relevant features from numerous variables and a limited number of cases was again demonstrated here as it performed well with all datasets.

Interestingly, the fact that the model used metabolites issued for all four settings of LC/MS (i.e., HILIC and C18 columns with both positive and negative ionization) showed the importance of multiplying the protocols to optimize the output of a metabolomics investigation. Of note, the dataset built only with metabolomics data did not show high levels of performance, highlighting the complementary nature of both sources of data.

Comparing our results on the clinical dataset alone with published investigations (references in Supplementary Table S1 and the Supplementary Synthetic Literature Review), we notice similarities in the variables demonstrating the highest weight in the model. However, other parameters are not represented in our model, such as immune cell balance, blood gas results, etc.; however, this may be explained by the fact that there is a lot of correlation between these parameters (Supplementary Figure S2). To our knowledge, this is the first study regrouping this number of clinical parameters in a single patient cohort and presenting this type of result, which could justify a reduction in the number of biochemical analyses performed in the future.

The level of performance reached by our algorithm (0.92) is higher than the results of other studies (Supplementary Table S1 and the Supplementary Synthetic Literature Review [18,24]) as well as on par with more recent algorithms such as the one combining plasma KL-6 and RSG [3]. It would thus be very interesting to combine these approaches in a larger-scale study.

The important variables of the full dataset models revealed a majority of metabolites, and preliminary attempts at identification showed sensible data in regard to the pathology (Supplementary Table S3). Interestingly, our findings are in line with a recent review which highlights the involvement of ceramides and other lipid mediators in COVID infection [12], although not in totality. This may be due to the differing aims between previous studies and ours: while others have been describing the metabolic profiles in the context of COVID infection [25], we aimed at predicting severity.

Of note, measuring the potential of the top 10 metabolites by PCA to discriminate between the groups (Supplementary Figure S3) demonstrated a good level of performance and the importance of interaction between the metabolites.

Our study remains limited by the sample size, rather small for a machine learning approach. However, in accordance with the TRIPOD guidelines, we showed that the risk of overfitting is low with a decoy strategy (Supplementary Figure S4), highlighting the specific nature of our models towards COVID severity in our patients. Our results are thus preliminary, but our goal was to demonstrate the added value of metabolomics and not necessarily to publish a defined predictive algorithm. An interesting perspective would be to test the complementarity of metabolomics with newer techniques such as FOT [2] or plasma KL-6 levels and RS) [3], which may be the object of a future study on a larger scale. A final limitation of the paper is the use of standard ML algorithms, and indeed, further studies would profit from the use of novel and innovative approaches [26,27]

While an important limitation in metabolomics study is the lack of inter-laboratory unification in terms of techniques and controls, recent efforts have been made towards standardization of the technique [28,29]. Hence, high throughput metabolomics has a very high potential for deployment in the clinic.

## 4. Materials and Methods

This work was performed following the TRIPOD guidelines (Supplementary Data) [30].

### 4.1. Patient Population

Patients were included at the Poitiers CHU (France) from 12-2020 to 4-2021 if they were hospitalized, their SARS-CoV-2-positive status was confirmed by RT-PCR, they were adults and free of any tutelage, they were affiliated to social security and gave informed consent. The patients included in the cohort were not vaccinated.

### 4.2. Judgment Criteria

Patients requiring mechanical ventilation were deemed to have a severe infection (45.31% of included patients, 29/64).

### 4.3. Clinical Parameters

Clinical parameters were chosen according to the literature (Supplementary Table S1). Biochemical analyses were performed on COBAS Pro automatons (Roche, Meylan, France). Samples were stored at the Poitiers CHU Biological Resources Center (BB-0033-00068).

### 4.4. Metabolomics Analysis

Procedures were performed in a blinded manner according to previously optimized protocol in which all the technical details are highlighted and the choices in extraction and liquid chromatography parameters are optimized [31]. The data were then pre-processed by the Compound Discoverer 3.3 software. Contributing variable names were imputed from interrogating MetaboLights, Metabolomics Workbench and Human Metabolome datasets [32] with the LC-HRMS signals.

### 4.5. Statistical Analysis and Machine Learning

To reduce interference from the LC-HRMS data, feature pre-selection was applied only for metabolites appearing to be different between the severe/non-severe groups (evaluated by *t*-test after parameter checks with F-test and Shapiro–Wilk test, p threshold set at 0.2) [33]. We used the R software 4.3.3 [34] with the tidyverse environment [35] to analyze the dataset created from clinical parameters and metabolomics screening. Missing data were minimal (1.2%) and were imputed using missRanger.

A standard training/testing set approach was adopted (80/20 split). The artificial intelligence classifiers were chosen according to the literature [36,37], as well as their technology, to test several ways of apprehending the data in order to extract the relevant information. We thus compared the performance of general logarithmic regression (GLM), K-nearest neighbor (KNN), support vector machine (SVM), Random Forest and boosted trees (C5.0). Candidates with the best performance (estimated by shape of the ROC curve, its AUC, the specificity, sensitivity, Brier Score and Youden’s Index) on the testing set of the data (i.e., not used for classifier training) were selected. Hyperparameter tuning was performed by 0.632 Bootstrapping. The risk of overfitting was estimated by performing the decoy strategy, in which the outcome variable was scrambled and the whole ML strategy was reperformed. The significance level was set at 0.05.

## 5. Conclusions

We demonstrate the highly relevant potential of metabolomics in studies aimed at the discovery of new biological signals and their inclusion into novel predictive algorithms. While our study was limited by the number of patients and was thus preliminary, we provided good evidence that combined with the power of machine learning, metabolomics can help design the mathematical models needed to advance towards personalized medicine. Moreover, unlike biological signals issued from other omics [38], the transfer of a metabolomics-based prediction algorithm to the healthcare professional does not present many hurdles and could rapidly improve patient care. Further development of metabolomics should include larger cohorts and more modern ML approaches to fully take advantages of the richness of the data produced.

## Figures and Tables

**Table 1:** Performance indicators for each of the predictive algorithms built with either the clinical and bloodwork dataset (top) or the full dataset which includes metabolites (middle) or both (bottom).

**Clinical and Bloodwork Dataset**						
	AUC (95%CI)	Specificity	Sensitivity	Brier Score	Youden’s index	[Acc > NIR] *p*-Value
GLM	0.62 (0.32, 0.86)	0.71	0.5	1.31	0.214	0.3938
RandomForest	0.85 (0.55, 0.98)	1	0.67	1.38	0.67	0.0222
KNN	0.77 (0.46, 0.95)	0.86	0.67	1.46	0.52	0.0798
SVM	0.69 (0.39, 0.91)	0.71	0.67	1.54	0.381	0.2033
C5.0	0.77 (0.46, 0.95)	0.71	0.83	1.77	0.55	0.0798
**Metabolomics Alone Dataset**						
	AUC (95%CI)	Specificity	Sensitivity	Brier Score	Youden’s index	[Acc > NIR] *p*-Value
GLM	0.7 (0.46, 0.88)	0.81	0.56	1.3	0.374	0.1299
RandomForest	0.75 (0.50, 0.91)	1	0.44	1.05	0.44	0.0553
KNN	0.7 (0.46, 0.88)	0.64	0.78	1.7	0.414	0.1299
SVM	0.8 (0.56, 0.94)	1	0.56	1.2	0.556	0.0189
C5.0	0.7 (0.46, 0.88)	1	0.33	0.9	0.33	0.1299
**Full Dataset Which Includes Metabolites**						
	AUC (95%CI)	Specificity	Sensitivity	Brier Score	Youden’s index	[Acc > NIR] *p*-Value
GLM	0.77 (0.46, 0.95)	0.86	0.67	1.46	0.52	0.0798
RandomForest	0.92 (0.64, 0.99)	1	0.83	1.62	0.83	0.0039
KNN	0.92 (0.64, 0.99)	0.86	1	1.92	0.857	0.0039
SVM	0.69 (0.39, 0.91)	1	0.64	0.923	0.333	0.9623
C5.0	0.77 (0.46, 0.95)	0.86	0.67	1.46	0.524	0.0798

Performance statistics were calculated with R.

## Data Availability

The data that support the findings of this study are attached as Supplementary Materials.

## Supplementary Materials

The following supporting information can be downloaded at: https://www.mdpi.com/article/10.3390/ijms252212199/s1. References [39,40,41,42,43,44,45,46,47,48,49,50,51,52,53] are cited in the Supplementary Materials.
