# Supplementary material for: Advantages of Metabolomics-Based Multivariate Machine Learning to Predict Disease Severity: Example of COVID
Source: Int J Mol Sci. 2024 Nov 13;25(22):12199. doi: 10.3390/ijms252212199 (PMC11594300; doi:10.3390/ijms252212199)
Supplement: Supplementary file 1 [file ijms-25-12199-s001.zip › Supp Fig 1.pptx]

## Slide 1
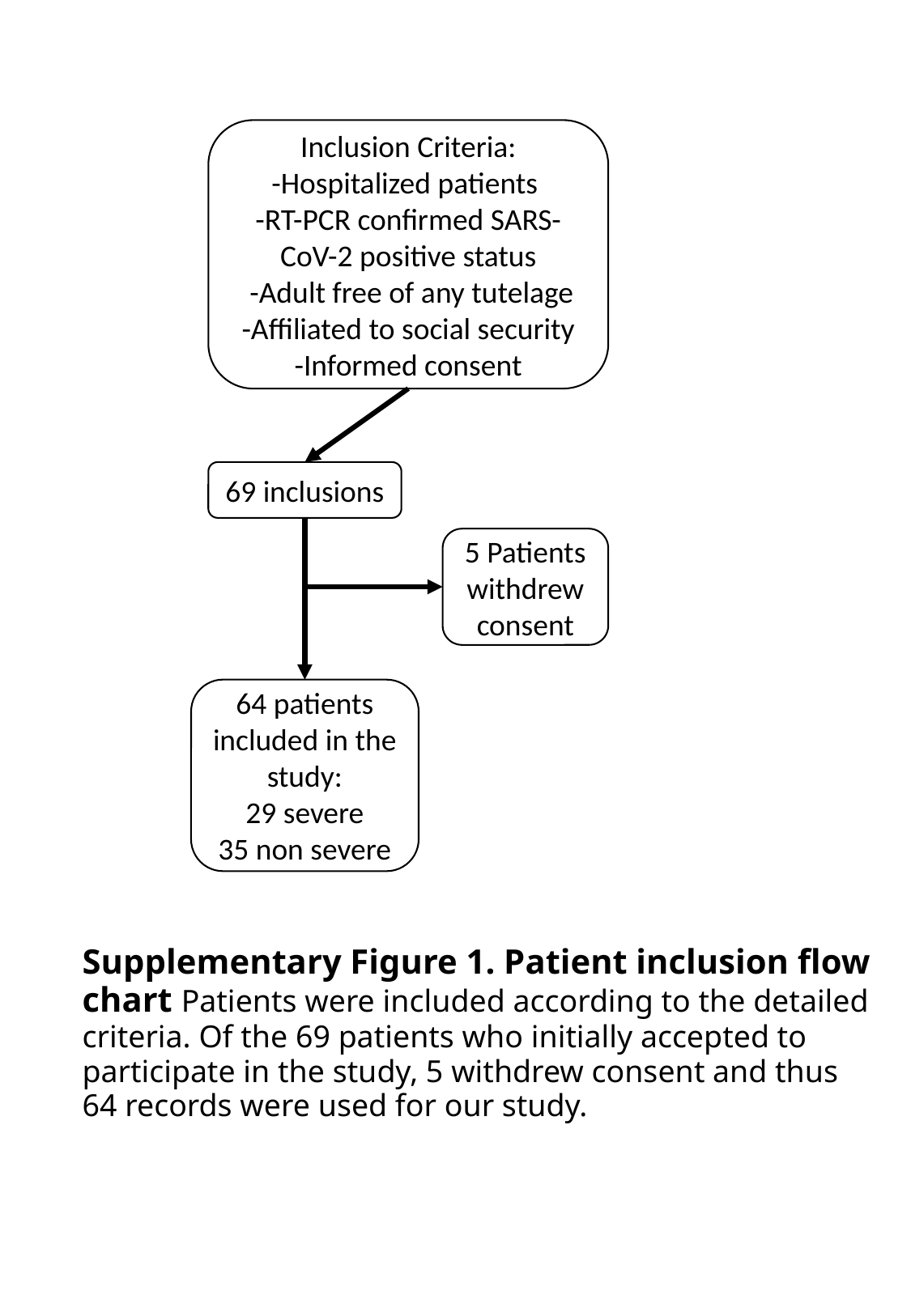

Inclusion Criteria:
-Hospitalized patients
-RT-PCR confirmed SARS-CoV-2 positive status
 -Adult free of any tutelage
-Affiliated to social security
-Informed consent
69 inclusions
5 Patients withdrew consent
64 patients included in the study:
29 severe
35 non severe
Supplementary Figure 1. Patient inclusion flow chart Patients were included according to the detailed criteria. Of the 69 patients who initially accepted to participate in the study, 5 withdrew consent and thus 64 records were used for our study.
