# Supplementary material for: Advantages of Metabolomics-Based Multivariate Machine Learning to Predict Disease Severity: Example of COVID
Source: Int J Mol Sci. 2024 Nov 13;25(22):12199. doi: 10.3390/ijms252212199 (PMC11594300; doi:10.3390/ijms252212199)
Supplement: Supplementary file 1 [file ijms-25-12199-s001.zip › Supp Fig 2.pptx]

## Slide 1
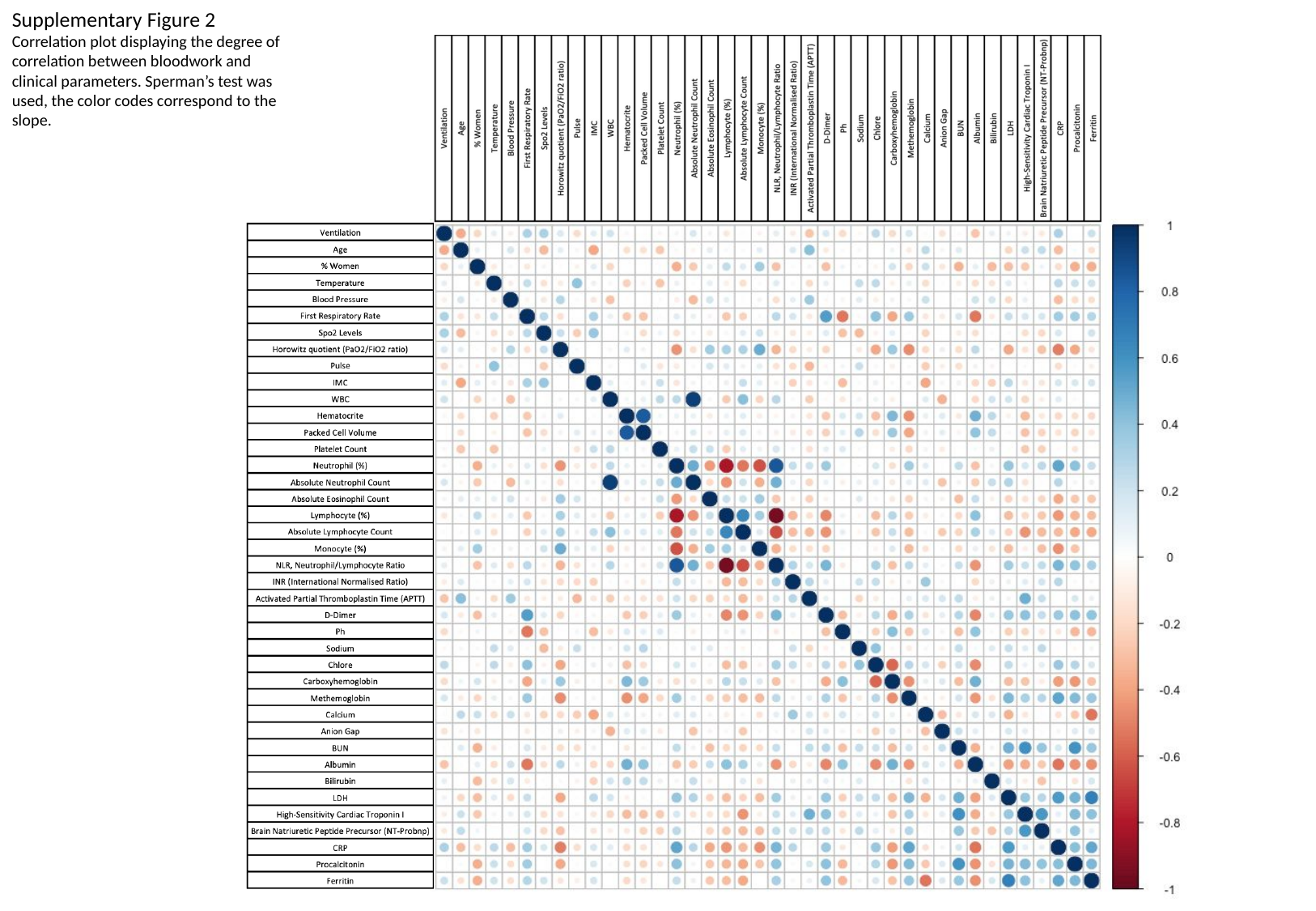

Supplementary Figure 2
Correlation plot displaying the degree of correlation between bloodwork and clinical parameters. Sperman’s test was used, the color codes correspond to the slope.
