# Supplementary material for: Advantages of Metabolomics-Based Multivariate Machine Learning to Predict Disease Severity: Example of COVID
Source: Int J Mol Sci. 2024 Nov 13;25(22):12199. doi: 10.3390/ijms252212199 (PMC11594300; doi:10.3390/ijms252212199)
Supplement: Supplementary file 1 [file ijms-25-12199-s001.zip › Supp Fig 3.pptx]

## Slide 1
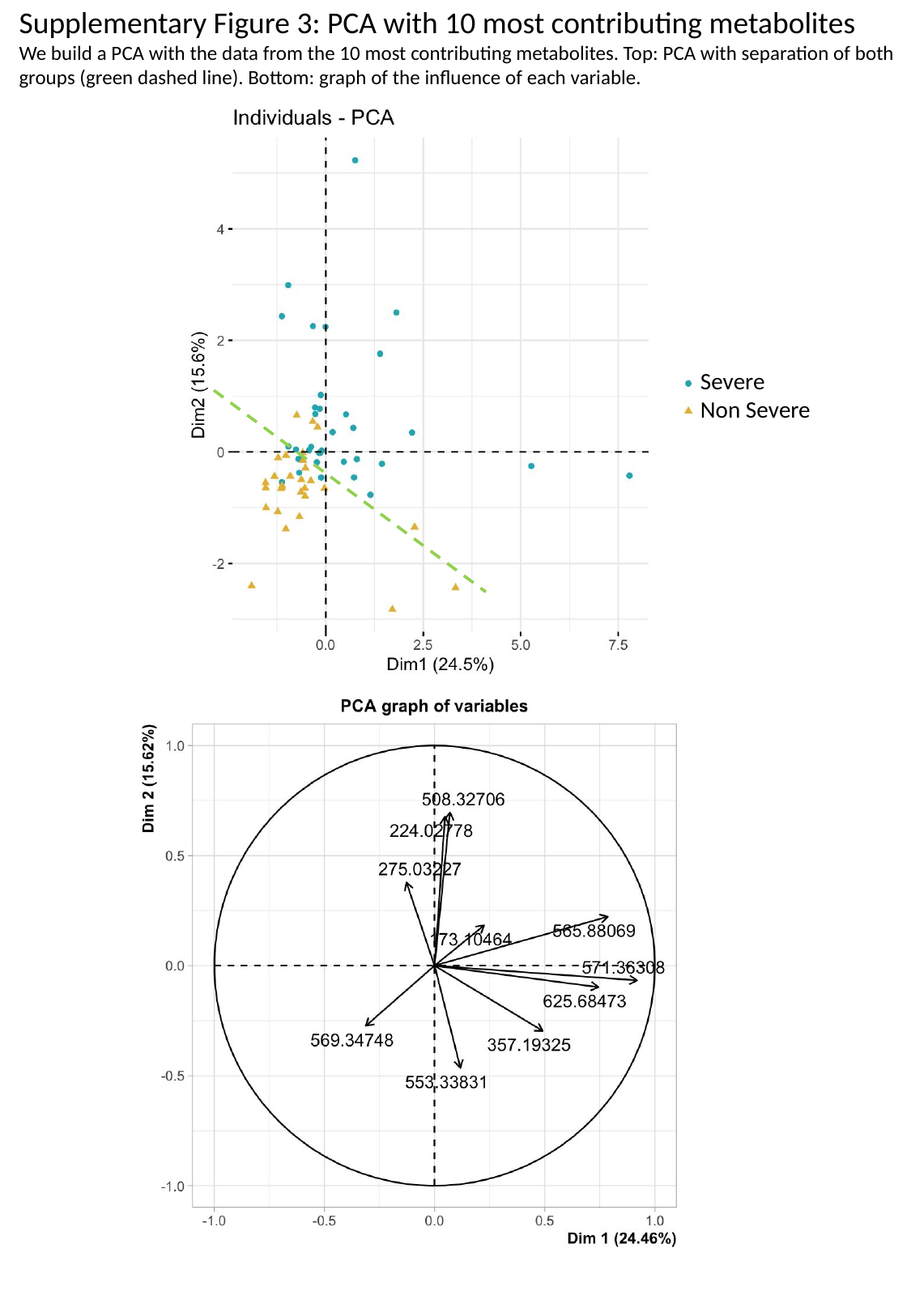

Supplementary Figure 3: PCA with 10 most contributing metabolites
We build a PCA with the data from the 10 most contributing metabolites. Top: PCA with separation of both groups (green dashed line). Bottom: graph of the influence of each variable.
Severe
Non Severe
