# Supplementary material for: Advantages of Metabolomics-Based Multivariate Machine Learning to Predict Disease Severity: Example of COVID
Source: Int J Mol Sci. 2024 Nov 13;25(22):12199. doi: 10.3390/ijms252212199 (PMC11594300; doi:10.3390/ijms252212199)
Supplement: Supplementary file 1 [file ijms-25-12199-s001.zip › Supp Fig 4.pptx]

## Slide 1
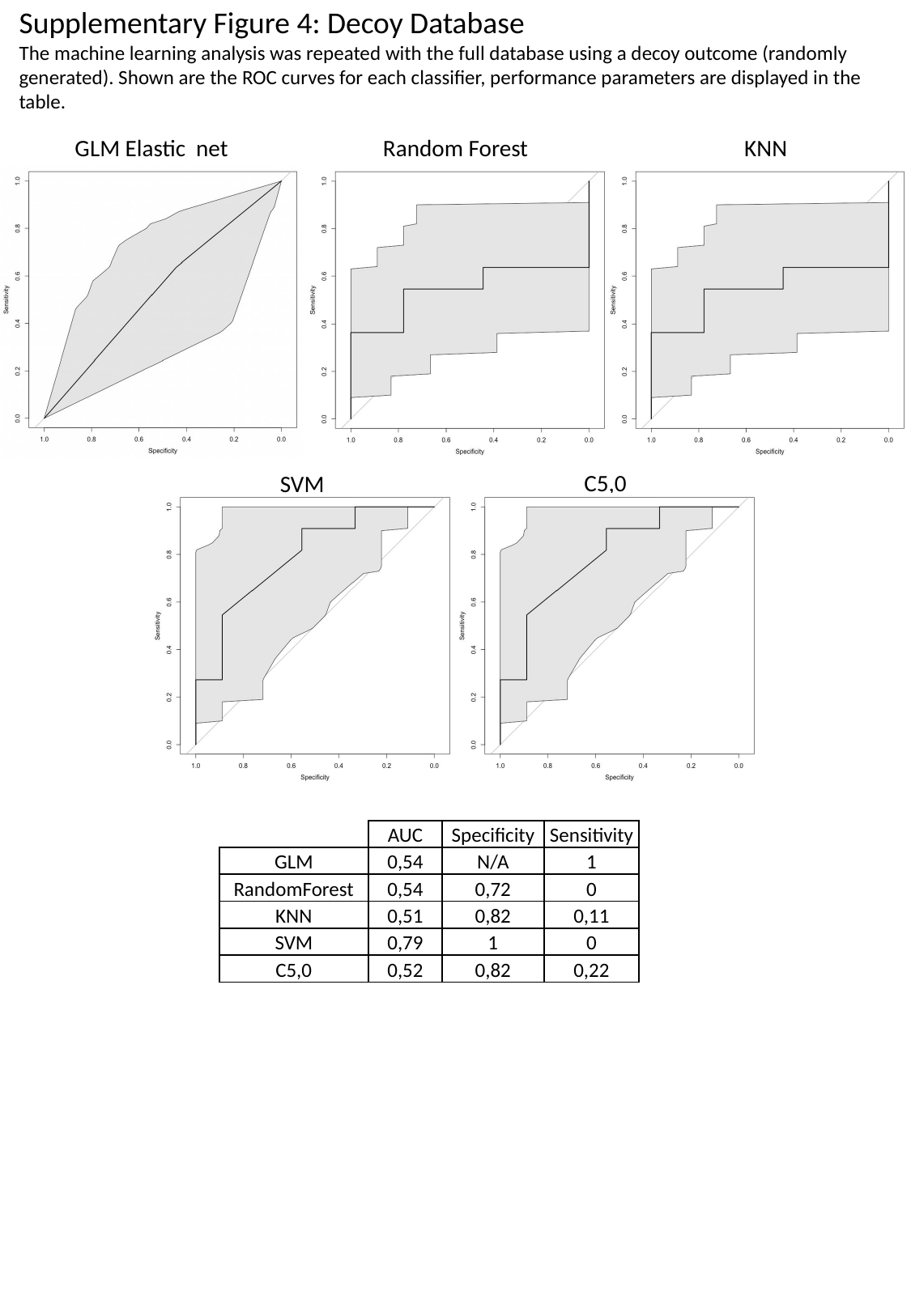

Supplementary Figure 4: Decoy Database
The machine learning analysis was repeated with the full database using a decoy outcome (randomly generated). Shown are the ROC curves for each classifier, performance parameters are displayed in the table.
GLM Elastic net
Random Forest
KNN
C5,0
SVM
| | AUC | Specificity | Sensitivity |
| --- | --- | --- | --- |
| GLM | 0,54 | N/A | 1 |
| RandomForest | 0,54 | 0,72 | 0 |
| KNN | 0,51 | 0,82 | 0,11 |
| SVM | 0,79 | 1 | 0 |
| C5,0 | 0,52 | 0,82 | 0,22 |
