# Supplementary material for: Advantages of Metabolomics-Based Multivariate Machine Learning to Predict Disease Severity: Example of COVID
Source: Int J Mol Sci. 2024 Nov 13;25(22):12199. doi: 10.3390/ijms252212199 (PMC11594300; doi:10.3390/ijms252212199)
Supplement: Supplementary file 1 [file ijms-25-12199-s001.zip › Supplementary Table 1.pdf]

Supplementary Table S1: List of explored clinical and bloodwork parameters and bibliographic references describing their potential to predict COVID severity.

Parameter	Non Critical Patients (Mean±SD)	Critical Patients (Mean±SD)	t-test p value	Reference
Age	71,97±17,32	62,66±9,51	0,009*	(1) (2, 3) (4) (5) (6) (7) (8)
% Women	40% (14/35)	19,4% (6/29)	0,094	(4) (7)
Temperature	37,17±0,83	37,34±0,73	0,362	(9) (7)
Blood Pressure	93,77±15,82	91,28±18,25	0,565	(9) (7)
First Respiratory Rate	20,47±3,74	24,89±6,38	0,005*	(9) (7)
Spo2 Levels	93,71±2,8	95,52±1,77	0,003*	(9) (4) (6) (7)
Horowitz quotient (PaO2/FiO2 ratio)	177,1±70,65	209,92±73,99	0,128	(10)
Pulse	77,4±14,89	71,41±11,31	0,073	(7)
IMC	29,45±6,02	31,23±6,61	0,287	(9) (7)
WBC	7,49±3,46	9,77±5,71	0,067	(9)
Hematocrite	12,47±2,76	12,71±2,25	0,698	(11) (1)
Packed Cell Volume	37,87±8,21	37,44±8,25	0,836	(9)
Platelet Count	243,54±95,38	234,83±85,64	0,704	(10)
Neutrophil (%)	77,78±9,38	77,78±11,5	0,999	(9) (1)
Absolute Neutrophil Count	6,03±3,37	6,59±2,65	0,479	(9)
Absolute Eosinophil Count	0,18±0,41	0,13±0,27	0,582	(9)
Lymphocyte (%)	14,13±8,23	13,06±9,31	0,648	(9) (1) (4)
Absolute Lymphocyte Count	0,88±0,39	1,22±1,49	0,275	(12) (8)
Monocyte (%)	6,8±3,69	5,93±2,12	0,261	(1)
NLR, Neutrophil/Lymphocyte Ratio	8,38±6,34	10,45±10,77	0,397	(13) (2) (14)
INR (International Normalised Ratio)	1,07±0,1	1,06±0,09	0,811	(9) (4)
Activated Partial Thromboplastin Time (APTT)	41,52±10,85	36,37±5,79	0,036*	(1)
D-Dimer	1867,88±3612,1	1338,08±808,85	0,419	(2) (10)
Ph	7,46±0,03	7,44±0,05	0,097	(11)
Sodium	134,17±3,5	133,86±4,98	0,822	(1)
Chlore	101,28±3,91	103,9±4,94	0,077	(1)
Carboxyhemoglobin	0,52±0,3	0,43±0,39	0,418	(11)

Methemoglobin	0,25±0,13	0,29±0,12	0,348	(11)
Calcium	1,15±0,06	1,16±0,08	0,591	(7)
Anion Gap	15,86±2,24	15,17±2,35	0,240	(7)
BUN	9,5±7,22	9,25±5,62	0,879	(6)
Albumin	35,18±3,76	32,72±3,47	0,017*	(1, 7)
Bilirubin	7±2,99	7,82±3,5	0,353	(14)
LDH	394,11±169,59	384,67±122,05	0,854	(9) (1) (12) (15) (16) (4) (5) (7) (14) (8)
High-Sensitivity Cardiac Troponin I	31,7±32,49	32,31±58,82	0,973	(1)
Brain Natriuretic Peptide Precursor (NT-Probnp)	1829,07±3428,19	4329,18±16923,97	0,555	(1)
CRP	79,88±92,95	78,21±47,07	0,932	(1, 12, 17) (2) (5)
Procalcitonin	0,36±0,59	0,38±0,76	0,927	(5)
Ferritin	1310,47±1409,25	1829,6±1637,79	0,360	(9) (4)

Clinical and bloodwork parameters recorded during the study. Shown are means±SD. Statistics were performed using the Student's t-test, * : $p<0,05$).

References :

1. Chowdhury, M. E. H., Rahman, T., Khandakar, A., Al-Madeed, S., Zughaier, S. M., Doi, S. A. R., Hassen, H., and Islam, M. T. (2021) An Early Warning Tool for Predicting Mortality Risk of COVID-19 Patients Using Machine Learning. *Cogn. Comput.* 1–16
2. Weng, Z., Chen, Q., Li, S., Li, H., Zhang, Q., Lu, S., Wu, L., Xiong, L., Mi, B., Liu, D., Lu, M., Yang, D., Jiang, H., Zheng, S., and Zheng, X. (2020) ANDC: an early warning score to predict mortality risk for patients with Coronavirus Disease 2019. *J. Transl. Med.* **18**, 328
3. Chen, R., Liang, W., Jiang, M., Guan, W., Zhan, C., Wang, T., Tang, C., Sang, L., Liu, J., Ni, Z., Hu, Y., Liu, L., Shan, H., Lei, C., Peng, Y., Wei, L., Liu, Y., Hu, Y., Peng, P., Wang, J., Liu, J., Chen, Z., Li, G., Zheng, Z., Qiu, S., Luo, J., Ye, C., Zhu, S., Liu, X., Cheng, L., Ye, F., Zheng, J., Zhang, N., Li, Y., He, J., Li, S., Zhong, N., and Medical Treatment Expert Group for COVID-19. (2020) Risk Factors of Fatal Outcome in Hospitalized Subjects With Coronavirus Disease 2019 From a Nationwide Analysis in China. *Chest* **158**, 97–105
4. Kar, S., Chawla, R., Haranath, S. P., Ramasubban, S., Ramakrishnan, N., Vaishya, R., Sibal, A., and Reddy, S. (2021) Multivariable mortality risk prediction using machine learning for COVID-19 patients at admission (AICOVID). *Sci. Rep.* **11**, 12801
5. Subudhi, S., Verma, A., Patel, A. B., Hardin, C. C., Khandekar, M. J., Lee, H., McEvoy, D., Stylianopoulos, T., Munn, L. L., Dutta, S., and Jain, R. K. (2021) Comparing machine learning algorithms for predicting ICU admission and mortality in COVID-19. *NPJ Digit. Med.* **4**, 87
6. Mahdavi, M., Choubdar, H., Zabeh, E., Rieder, M., Safavi-Naeini, S., Jobbagy, Z., Ghorbani, A., Abedini, A., Kiani, A., Khanlarzadeh, V., Lashgari, R., and Kamrani, E. (2021) A machine learning based exploration of COVID-19 mortality risk. *PloS One* **16**, e0252384

7. Hao, B., Sotudian, S., Wang, T., Xu, T., Hu, Y., Gaitanidis, A., Breen, K., Velmahos, G. C., and Paschalidis, I. C. (2020) Early prediction of level-of-care requirements in patients with COVID-19. *eLife* **9**, e60519
8. Ji, D., Zhang, D., Xu, J., Chen, Z., Yang, T., Zhao, P., Chen, G., Cheng, G., Wang, Y., Bi, J., Tan, L., Lau, G., and Qin, E. (2020) Prediction for Progression Risk in Patients With COVID-19 Pneumonia: The CALL Score. *Clin. Infect. Dis. Off. Publ. Infect. Dis. Soc. Am.* **71**, 1393–1399
9. Alle, S., Kanakan, A., Siddiqui, S., Garg, A., Karthikeyan, A., Mehta, P., Mishra, N., Chattopadhyay, P., Devi, P., Waghdhare, S., Tyagi, A., Tarai, B., Hazarik, P. P., Das, P., Budhiraja, S., Nangia, V., Dewan, A., Sethuraman, R., Subramanian, C., Srivastava, M., Chakravarthi, A., Jacob, J., Namagiri, M., Konala, V., Dash, D., Sethi, T., Jha, S., Agrawal, A., Pandey, R., Vinod, P. K., and Priyakumar, U. D. (2022) COVID-19 Risk Stratification and Mortality Prediction in Hospitalized Indian Patients: Harnessing clinical data for public health benefits. *PloS One* **17**, e0264785
10. Magunia, H., Lederer, S., Verbuecheln, R., Gilot, B. J., Koeppen, M., Haeberle, H. A., Mirakaj, V., Hofmann, P., Marx, G., Bickenbach, J., Nohe, B., Lay, M., Spies, C., Edel, A., Schiefenhövel, F., Rahmel, T., Putensen, C., Sellmann, T., Koch, T., Brandenburger, T., Kindgen-Milles, D., Brenner, T., Berger, M., Zacharowski, K., Adam, E., Posch, M., Moerer, O., Scheer, C. S., Sedding, D., Weigand, M. A., Fichtner, F., Nau, C., Prätsch, F., Wiesmann, T., Koch, C., Schneider, G., Lahmer, T., Straub, A., Meiser, A., Weiss, M., Jungwirth, B., Wappler, F., Meybohm, P., Herrmann, J., Malek, N., Kohlbacher, O., Biergans, S., and Rosenberger, P. (2021) Machine learning identifies ICU outcome predictors in a multicenter COVID-19 cohort. *Crit. Care Lond. Engl.* **25**, 295
11. Huyut, M. T. and Üstündağ, H. (2022) Prediction of diagnosis and prognosis of COVID-19 disease by blood gas parameters using decision trees machine learning model: a retrospective observational study. *Med. Gas Res.* **12**, 60–66
12. Yan, L., Zhang, H.-T., Goncalves, J., Xiao, Y., Wang, M., Guo, Y., Sun, C., Tang, X., Jing, L., Zhang, M., Huang, X., Xiao, Y., Cao, H., Chen, Y., Ren, T., Wang, F., Xiao, Y., Huang, S., Tan, X., Huang, N., Jiao, B., Cheng, C., Zhang, Y., Luo, A., Mombaerts, L., Jin, J., Cao, Z., Li, S., Xu, H., and Yuan, Y. (2020) An interpretable mortality prediction model for COVID-19 patients. *Nat. Mach. Intell.* **2**, 283–288
13. Liu, J., Liu, Y., Xiang, P., Pu, L., Xiong, H., Li, C., Zhang, M., Tan, J., Xu, Y., Song, R., Song, M., Wang, L., Zhang, W., Han, B., Yang, L., Wang, X., Zhou, G., Zhang, T., Li, B., Wang, Y., Chen, Z., and Wang, X. (2020) Neutrophil-to-lymphocyte ratio predicts critical illness patients with 2019 coronavirus disease in the early stage. *J. Transl. Med.* **18**, 206
14. Liang, W., Liang, H., Ou, L., Chen, B., Chen, A., Li, C., Li, Y., Guan, W., Sang, L., Lu, J., Xu, Y., Chen, G., Guo, H., Guo, J., Chen, Z., Zhao, Y., Li, S., Zhang, N., Zhong, N., He, J., and China Medical Treatment Expert Group for COVID-19. (2020) Development and Validation of a Clinical Risk Score to Predict the Occurrence of Critical Illness in Hospitalized Patients With COVID-19. *JAMA Intern. Med.* **180**, 1081–1089
15. Yan, L., Zhang, H.-T., Goncalves, J., Xiao, Y., Wang, M., Guo, Y., Sun, C., Tang, X., Jin, L., Zhang, M., Huang, X., Xiao, Y., Cao, H., Chen, Y., Ren, T., Wang, F., Xiao, Y., Huang, S., Tan, X., Huang, N., Jiao, B., Zhang, Y., Luo, A., Mombaerts, L., Jin, J., Cao, Z., Li, S., Xu, H., and Yuan, Y. (2020) A machine learning-based model for survival prediction in patients with severe COVID-19 infection. 2020.02.27.20028027
16. Kishaba, T., Tamaki, H., Shimaoka, Y., Fukuyama, H., and Yamashiro, S. (2014) Staging of acute exacerbation in patients with idiopathic pulmonary fibrosis. *Lung* **192**, 141–149

17. Lu, J., Hu, S., Fan, R., Liu, Z., Yin, X., Wang, Q., Lv, Q., Cai, Z., Li, H., Hu, Y., Han, Y., Hu, H., Gao, W., Feng, S., Liu, Q., Li, H., Sun, J., Peng, J., Yi, X., Zhou, Z., Guo, Y., and Hou, J. (2020) ACP risk grade: a simple mortality index for patients with confirmed or suspected severe acute respiratory syndrome coronavirus 2 disease (COVID-19) during the early stage of outbreak in Wuhan, China. 2020.02.20.20025510
