# Supplementary material for: Advantages of Metabolomics-Based Multivariate Machine Learning to Predict Disease Severity: Example of COVID
Source: Int J Mol Sci. 2024 Nov 13;25(22):12199. doi: 10.3390/ijms252212199 (PMC11594300; doi:10.3390/ijms252212199)
Supplement: Supplementary file 1 [file ijms-25-12199-s001.zip › Supplementary Table 3.pdf]

Supplementary Table S3: Top 10 contributing parameters to the Random Forest model built from the full dataset.

Rank	Technique	Monoisotopic mass	Putative identification
1	HILIC ESI+	357,19325	Laudanosine (HMDB0030213), 5-hydroxypropafenone (HMDB0060988) or Nalbuphine (HMDB0014982)
2	HILIC ESI+	565,88069	Cer(d18:0/18:1(9Z)) (HMDB0011763)
3	HILIC ESI+	625,68473	Cer(d18:1/22:5(4Z,7Z,10Z,13Z,19Z)-O(16,17)) (HMDB0290036)
4	HILIC ESI+	173,10464	Isovalerylalanine (HMDB0000747), Hexanoylglycine (HMDB0000701), isocaproylglycine (HMDB0341334) or N-Acetylisoleucine (HMDB0061684)
5	HILIC ESI+	508,32706	PA(8:0/i-14:0) (HMDB0115687)
6	C18 ESI+	569,34748	LysoPC(22:5(4Z,7Z,10Z,13Z,16Z)/0:0) (HMDB0010402) or LysoPC(22:5(7Z,10Z,13Z,16Z,19Z)/0:0) (HMDB0010403)
7	C18 ESI-	571,36308	LysoPC(22:4(7Z,10Z,13Z,16Z)/0:0) (HMDB0010401)
8	HILIC ESI-	275,03227	N-(2-formyl-3-chlorophenyl)anthranilic acid (HMDB0060006)
9	HILIC ESI-	553,33831	LysoPE(24:6(6Z,9Z,12Z,15Z,18Z,21Z)/0:0) (HMDB0011529)
10	HILIC ESI-	224,02778	Cysteinyl-Cysteine (HMDB0028772)

The first metabolite (possible analog to Laudanosine, 5-hydroxypropafenone, Nalbuphine) is likely involved in analgesia, a logical response to severe disease. The second and third are ceramides, members of the sphingolipids class, which have a large panel of activities and demonstrated impaired metabolism in several pathologies such as diabetes, various cancers, microbial infections, diseases of the cardiovascular and respiratory systems, Alzheimer's disease and other neurological syndromes [36]. Variables 6, 7 and 9 are lysophospholipids, lipid mediators that exert their diverse pathophysiological functions via G protein-coupled receptors and which heir levels have been found to dramatically increase during various pathological conditions [37]. Hence, the nature of the metabolites contributed to the COVID-severity prediction model are known signaling molecules already described in pathological contexts.
