# Supplementary material for: Advantages of Metabolomics-Based Multivariate Machine Learning to Predict Disease Severity: Example of COVID
Source: Int J Mol Sci. 2024 Nov 13;25(22):12199. doi: 10.3390/ijms252212199 (PMC11594300; doi:10.3390/ijms252212199)
Supplement: Supplementary file 1 [file ijms-25-12199-s001.zip › Synthetic litterature review.pdf]

Synthetic literature review

Prediction using clinical parameters:

- Hao et al [1] developed predictive models to forecast the need for hospitalization, ICU admission, and mechanical ventilation in COVID-19 patients based on patient demographics, symptoms, medications, and comorbidities, while the ICU and ventilation models also used lab results and imaging findings. The models achieving high accuracy (AUC 86-88%) and outperforming standard pneumonia severity scores.

- Liang et al [2] described the development and validation of a clinical risk score, called COVID-GRAM, includes 10 variables that were identified as independent predictors of critical illness, to predict the occurrence of critical illness in hospitalized patients with COVID-19 : X-ray abnormality, Age, Hemoptysis, Dyspnea, Unconsciousness, Comorbidities, Cancer history, Neutrophil-lymphocyte ratio, Lactate dehydrogenase, Direct bilirubin. The COVID-GRAM risk score outperformed the CURB-6 model, which is commonly used to assess the severity of community-acquired pneumonia.

- Liu et al [3] found that older COVID-19 patients had more severe clinical manifestations, greater disease severity, and longer disease courses compared to younger patients. They determined that older patients had more severe laboratory abnormalities, including higher levels of blood urea nitrogen, lactate dehydrogenase, and inflammatory markers, compared to younger patients.

- Rudd et al [4] conducted a systematic review that examines the current literature on machine learning models for the diagnosis and prognosis of COVID-19 using chest radiographs and CT scans, and finds that none of the reviewed models are ready for clinical use due to methodological flaws and biases.

- Subudhi et al [5] evaluated 18 different machine learning algorithms to predict ICU admission and mortality in COVID-19 patients, and found that ensemble-based models performed the best, with key predictors including C-reactive protein, lactate dehydrogenase, oxygen saturation, and kidney function. Specific clinical and laboratory variables like CRP, LDH, neutrophil/lymphocyte percentages, oxygen saturation, ventilator use, and kidney function were important predictors of ICU admission and mortality. The importance of some variables like D-dimer and oxygen saturation changed when validating the models on a temporally distinct patient cohort, suggesting changes in COVID-19 management over time.

- Chowdhury et al [6] provided a simple, easy-to-use and reliable scoring system using Lactate dehydrogenase, neutrophils (%), lymphocyte (%), high-sensitivity C-reactive protein, and age for the prognosis of risk severity of individuals suffering from COVID-19 to stratify them into appropriate risk groups and provide them necessary health support accordingly.

- Kar et al [7] developed and validated a machine learning model to predict mortality risk in COVID-19 patients at the time of admission using clinical and laboratory parameters (including age, gender, respiratory symptoms, comorbidities, and inflammatory markers), and the model achieved high accuracy in both the development and validation cohorts (AUC of 0.782 and accuracy of 0.93 in the validation cohort).

- Mahdavi et al [8] developed and compared machine learning models for predicting COVID-19 mortality risk using invasive laboratory and non-invasive clinical and demographic data from patients' first day of admission, and finds that non-invasive features can provide mortality predictions similar to invasive features and the joint model, suggesting several non-invasive

features like SPO2 and age contain significant predictive information for COVID-19 mortality.

- Magunia et al [9] developed a machine learning model to predict ICU outcomes and identified key predictors of ICU survival, ECMO therapy, and renal replacement therapy in COVID-19 patients. The most important predictors of ICU survival were age, platelet/neutrophil ratio, D-dimer levels, and ARDS severity.

- Alle et al [10] analyzed clinical data from a cohort of Indian COVID-19 hospitalized patients to develop risk stratification and mortality prediction models using CRP, LDH, neutrophil, lymphocyte and age, comparing the Indian cohort to the Wuhan cohort, and analyzes the role of steroids in patient outcomes.

- Huyut and Üstündağ [11] determined the diagnosis and prognosis of COVID-19 disease using blood gas parameters and a machine learning decision tree model (CHAID). They showed that low ionized calcium significantly predicted the need for intensive care of COVID-19 patients and that low carboxyhemoglobin, high pH, low sodium, low hematocrit and low methemoglobin were significant independent markers in detecting COVID-19.

Prediction using metabolomics:

- Fricke-Galindo et al [12] provided a comprehensive review of the application of metabolomics in the diagnosis and prognosis of COVID-19. They highlighted that metabolic pathways related to amino acids, lipids, and energy were affected in severe COVID-19 cases, with the tryptophan-kynurenine pathway being consistently dysregulated, indicating potential prognostic value of metabolites like tryptophan, kynurenine, and 3-hydroxykynurenine.

- Sharif-Askari et al [13] investigated the utility of saliva and plasma metabolomic profiles as potential parameters for risk stratifying COVID-19 patients into asymptomatic, mild, moderate, and severe groups. They showed that sphingosine, kynurenine, linoleic acid, and Alpha-ketoisovaleric acid were associated with COVID-19 severity.

- Gikas et al [14] attempted to identify plasma metabolomic signatures that can be used for the diagnosis and prognosis of COVID-19 severity, and to provide insights into the underlying biological mechanisms of the disease. They highlight that changes in plasma levels of lipids and amino acids, particularly higher levels of unsaturated lipids and fructosyl amino acids and lower levels of saturated lipids, serve as robust biomarkers for SARS-CoV-2 infection and COVID-19 severity.

- Battaglini et al [15] provided a comprehensive review of laboratory biomarkers such as These include interleukin (IL)-1b, IL-2, IL-6, IL-8, interferon (IFN)-g-induced protein 10, granulocyte colony-stimulating factor, monocyte chemoattractant protein 1, macrophage inflammatory protein-1a, and tumor necrosis factor-a; that have shown significant diagnostic and prognostic value for risk stratification in COVID-19, and discussed the potential clinical application of novel analytic strategies such as metabolomics and proteomics.

- Bruzzone et al [16]also compiled a comprehensive review of the use of metabolomics to study various aspects of COVID-19, including the characteristic metabolic signature, the ability to discriminate patients based on disease severity, the effects of drugs and vaccines, and the characterization of the natural history of the metabolic changes from infection to recovery or long-term sequelae.

- Frampas et al [17] describes an untargeted saliva metabolomics study that identified a panel of 6 metabolites (5 amino acids and 1 unidentified compound), used in a PLS-DA model which was able to correctly classify 97% of COVID-19 negative participants as "low risk", indicating the metabolic changes were specific to high severity COVID-19 and not just general poor health.

- Bourgin et al [18] provided a comprehensive overview of the use of mass spectrometric metabolomics to identify diagnostic, prognostic, and mechanistic biomarkers of COVID-19. They identify tryptophan metabolites, amino acids, vitamin B3, and lipid metabolites as likely to contribute to the excessive inflammation and impaired immune response seen in severe COVID-19.

Bibliography:

1. Hao, B.; Sotudian, S.; Wang, T.; Xu, T.; Hu, Y.; Gaitanidis, A.; Breen, K.; Velmahos, G.C.; Paschalidis, I.C. Early Prediction of Level-of-Care Requirements in Patients with COVID-19. *Elife* **2020**, *9*, e60519, doi:10.7554/eLife.60519.
2. Liang, W.; Liang, H.; Ou, L.; Chen, B.; Chen, A.; Li, C.; Li, Y.; Guan, W.; Sang, L.; Lu, J.; et al. Development and Validation of a Clinical Risk Score to Predict the Occurrence of Critical Illness in Hospitalized Patients With COVID-19. *JAMA Intern Med* **2020**, *180*, 1081–1089, doi:10.1001/jamainternmed.2020.2033.
3. Liu, Y.; Mao, B.; Liang, S.; Yang, J.-W.; Lu, H.-W.; Chai, Y.-H.; Wang, L.; Zhang, L.; Li, Q.-H.; Zhao, L.; et al. Association between Age and Clinical Characteristics and Outcomes of COVID-19. *Eur Respir J* **2020**, *55*, 2001112, doi:10.1183/13993003.01112-2020.
4. Roberts, M.; Driggs, D.; Thorpe, M.; Gilbey, J.; Yeung, M.; Ursprung, S.; Aviles-Rivero, A.I.; Etmann, C.; McCague, C.; Beer, L.; et al. Common Pitfalls and Recommendations for Using Machine Learning to Detect and Prognosticate for COVID-19 Using Chest Radiographs and CT Scans. *Nat Mach Intell* **2021**, *3*, 199–217, doi:10.1038/s42256-021-00307-0.
5. Subudhi, S.; Verma, A.; Patel, A.B.; Hardin, C.C.; Khandekar, M.J.; Lee, H.; McEvoy, D.; Stylianopoulos, T.; Munn, L.L.; Dutta, S.; et al. Comparing Machine Learning Algorithms for Predicting ICU Admission and Mortality in COVID-19. *NPJ Digit Med* **2021**, *4*, 87, doi:10.1038/s41746-021-00456-x.
6. Chowdhury, M.E.H.; Rahman, T.; Khandakar, A.; Al-Madeed, S.; Zughaier, S.M.; Doi, S.A.R.; Hassen, H.; Islam, M.T. An Early Warning Tool for Predicting Mortality Risk of COVID-19 Patients Using Machine Learning. *Cognit Comput* **2021**, 1–16, doi:10.1007/s12559-020-09812-7.
7. Kar, S.; Chawla, R.; Haranath, S.P.; Ramasubban, S.; Ramakrishnan, N.; Vaishya, R.; Sibal, A.; Reddy, S. Multivariable Mortality Risk Prediction Using Machine Learning for COVID-19 Patients at Admission (AICOVID). *Sci Rep* **2021**, *11*, 12801, doi:10.1038/s41598-021-92146-7.
8. Mahdavi, M.; Choubdar, H.; Zabeh, E.; Rieder, M.; Safavi-Naeini, S.; Jobbagy, Z.; Ghorbani, A.; Abedini, A.; Kiani, A.; Khanlarzadeh, V.; et al. A Machine Learning Based Exploration of COVID-19 Mortality Risk. *PLoS One* **2021**, *16*, e0252384, doi:10.1371/journal.pone.0252384.
9. Magunia, H.; Lederer, S.; Verbuecheln, R.; Gilot, B.J.; Koeppen, M.; Haeberle, H.A.; Mirakaj, V.; Hofmann, P.; Marx, G.; Bickenbach, J.; et al. Machine Learning Identifies ICU Outcome Predictors in a Multicenter COVID-19 Cohort. *Crit Care* **2021**, *25*, 295, doi:10.1186/s13054-021-03720-4.
10. Alle, S.; Kanakan, A.; Siddiqui, S.; Garg, A.; Karthikeyan, A.; Mehta, P.; Mishra, N.; Chattopadhyay, P.; Devi, P.; Waghdhare, S.; et al. COVID-19 Risk Stratification and Mortality Prediction in Hospitalized Indian Patients: Harnessing Clinical Data for Public Health Benefits. *PLoS One* **2022**, *17*, e0264785, doi:10.1371/journal.pone.0264785.
11. Huyut, M.T.; Üstündağ, H. Prediction of Diagnosis and Prognosis of COVID-19 Disease by Blood Gas Parameters Using Decision Trees Machine Learning Model: A Retrospective Observational Study. *Med Gas Res* **2022**, *12*, 60–66, doi:10.4103/2045-9912.326002.

12. Hasan, M.R.; Suleiman, M.; Pérez-López, A. Metabolomics in the Diagnosis and Prognosis of COVID-19. *Front Genet* **2021**, *12*, 721556, doi:10.3389/fgene.2021.721556.
13. Saheb Sharif-Askari, N.; Soares, N.C.; Mohamed, H.A.; Saheb Sharif-Askari, F.; Alsayed, H.A.H.; Al-Hroub, H.; Salameh, L.; Osman, R.S.; Mahboub, B.; Hamid, Q.; et al. Saliva Metabolomic Profile of COVID-19 Patients Associates with Disease Severity. *Metabolomics* **2022**, *18*, 81, doi:10.1007/s11306-022-01936-1.
14. Occelli, C.; Guigonis, J.-M.; Lindenthal, S.; Cagnard, A.; Graslin, F.; Brglez, V.; Seitz-Polski, B.; Dellamonica, J.; Levraut, J.; Pourcher, T. Untargeted Plasma Metabolomic Fingerprinting Highlights Several Biomarkers for the Diagnosis and Prognosis of Coronavirus Disease 19. *Front Med (Lausanne)* **2022**, *9*, 995069, doi:10.3389/fmed.2022.995069.
15. Battaglini, D.; Lopes-Pacheco, M.; Castro-Faria-Neto, H.C.; Pelosi, P.; Rocco, P.R.M. Laboratory Biomarkers for Diagnosis and Prognosis in COVID-19. *Front Immunol* **2022**, *13*, 857573, doi:10.3389/fimmu.2022.857573.
16. Bruzzone, C.; Conde, R.; Embade, N.; Mato, J.M.; Millet, O. Metabolomics as a Powerful Tool for Diagnostic, Pronostic and Drug Intervention Analysis in COVID-19. *Front Mol Biosci* **2023**, *10*, 1111482, doi:10.3389/fmolb.2023.1111482.
17. Frampas, C.F.; Longman, K.; Spick, M.; Lewis, H.-M.; Costa, C.D.S.; Stewart, A.; Dunn-Walters, D.; Greener, D.; Evetts, G.; Skene, D.J.; et al. Untargeted Saliva Metabolomics by Liquid Chromatography-Mass Spectrometry Reveals Markers of COVID-19 Severity. *PLoS One* **2022**, *17*, e0274967, doi:10.1371/journal.pone.0274967.
18. Bourgin, M.; Durand, S.; Kroemer, G. Diagnostic, Prognostic and Mechanistic Biomarkers of COVID-19 Identified by Mass Spectrometric Metabolomics. *Metabolites* **2023**, *13*, 342, doi:10.3390/metabo13030342.
